# Antibiotic duration and changes in FEV_1_ are not associated with time until next exacerbation in adult cystic fibrosis: a single center study

**DOI:** 10.1186/s12890-017-0503-6

**Published:** 2017-11-29

**Authors:** Julia C. Espel, Hannah L. Palac, Joanne F. Cullina, Alexandria P. Clarke, Susanna A. McColley, Michelle H. Prickett, Manu Jain

**Affiliations:** 1 Division of Pulmonary and Critical Care Medicine, Department of Medicine, Feinberg School of Medicine, Northwestern University, 240 E. Huron Ave., McGaw Mezzanine, Chicago, IL 60611 USA; 2 Department of Preventive Medicine, Northwestern University, Chicago, IL USA; 3 Lurie Children’s Hospital, Chicago, IL USA

**Keywords:** Antibiotics, Bronchiectasis, Cystic fibrosis, Exacerbation, Outcomes Pseudomonas aeruginosa

## Abstract

**Background:**

Pulmonary exacerbations (PEx) are a major driver of morbidity and mortality in cystic fibrosis and reducing their frequency by extending the time between them is an important therapeutic goal. Although treatment decisions for exacerbations are often made based on dynamic changes in lung function, it is not clear if these changes truly impact future exacerbation risk. We analyzed adults with chronic *Pseudomonas aeruginosa* infection to determine whether changes in FEV_1_ or duration of intravenous antibiotic therapy were associated with time to the next pulmonary exacerbation.

**Methods:**

Medical records and Cystic Fibrosis Foundation Patient Registry data were examined retrospectively to assess whether various patient-specific demographic factors and exacerbation-specific characteristics were associated with time until next exacerbation using the Andersen-Gill model in order to control for previous exacerbation frequency history.

**Results:**

We examined 59 patients with 221 CF pulmonary exacerbations over a 3-year study period. Mean age was 28.2 years and mean baseline FEV_1_ was 62% predicted. In our univariable model, fall in FEV_1_ at onset of exacerbation (median absolute −3% predicted change), recovery of FEV_1_ with treatment (median absolute +3% predicted change) and duration of IV antibiotics (median 16 days) were not associated with time to next exacerbation (median 93.5 days). Paradoxically each one-year increase in age was associated with a reduction in hazard of PEx by 3% (HR 0.97, *P* = 0.03, 95% CI 0.95–1.00).

**Conclusions:**

FEV1 drop and recovery associated with onset and treatment of a CF pulmonary exacerbation or duration of intravenous antibiotics were not predictive of time until next exacerbation. Our finding that older age may be associated with decreased hazard of exacerbation is likely due to a healthy survivor effect and should be controlled for in clinical trials of pulmonary exacerbations.

## Background

Morbidity and mortality in Cystic Fibrosis (CF) is primarily related to the severity of pulmonary disease and patients’ forced expiratory volume in 1 s (FEV_1_) declines annually by an average of 1%–2% [1]. Bronchiectasis can occur early in life and develops from a cycle of airway infection, inflammation and mucus obstruction. Punctuating this chronic progressive process is the periodic development of CF pulmonary exacerbations. While there are no consensus criteria defining a CF pulmonary exacerbation, these events consist of acute or subacute worsening of respiratory and/or systemic symptoms that may include dyspnea, cough, sputum production, fatigue, anorexia or weight loss, which are often associated with a fall in FEV_1_ [2].

Pulmonary exacerbations (PEx) induce significant morbidity in patients, including more rapid lung function decline [3–5], failure to recover to previous FEV_1_ baseline [6], a loss in quality of life [7, 8], and an increased risk of death and lung transplantation [4]. In addition there is risk of acute and cumulative toxicity of repeated antibiotic exposures used for the treatment of PEx. Thus prevention of future PEx is an important therapeutic goal, both with new treatments [9] and in the treatment of an individual PEx [10, 11]. PEx are generally managed by increasing the intensity of airway clearance treatments, oral or intravenous antibiotics, and other supportive measures [12, 13].

Studies assessing demographic and physiologic risk factors for PEx in CF patients have identified several variables. One group demonstrated that a lower baseline FEV_1_, chronic *Pseudomonas aeruginosa* infection and CF-related diabetes mellitus (CFRD) were associated with more frequent exacerbations in adults [14]. A cohort study from the United Kingdom suggested that older age was associated with a shorter interval between PEx [15]. More recent analyses have suggested that the strongest risk factor for a shorter time to future exacerbation is a higher number of exacerbations in the preceding year [16, 17]. Other factors associated with increased frequency of PEx included use of selected chronic CF therapies which may reflect the severity of the underlying lung disease [16].

When treating PEx in CF, important therapeutic goals include symptom relief and recovery of FEV_1_. An important additional goal is the prevention of future PEx because of their short-term and cumulative impact on quality of life, antibiotic adverse events, induction of bacterial resistance and mortality [4, 18, 19]. Two key decisions in treating PEx are the selection of antibiotics and determining the duration of antibiotic treatment. Clinicians often use recovery of lung function (typically measured as FEV_1_) as a therapeutic endpoint in selecting duration of antibiotic treatment despite a lack evidence for this practice or strong guidance from treatment guidelines [20, 21]. Further, the relationship between fall and recovery in FEV_1_ and the duration of intravenous antibiotics to future PEx risk is not well understood, and the data that exist are conflicting [16, 17, 22]. Thus we conducted analyses of PEx at our CF center to determine whether duration of intravenous antibiotics or FEV_1_ fall and recovery to baseline were associated with time until next exacerbation (TUNE) in adult CF patients with chronic *Pseudomonas aeruginosa* infection.

## Methods

### Data collection

We examined retrospectively a cohort of adult CF patients followed at our center over a 3 year period using data obtained from our electronic medical record and from the Cystic Fibrosis Foundation Patient Registry (CFFPR). All patients who were enrolled in the study had given consent for enrolling in the CFFPR which gives permission for observational research using their data. We included all registry patients with a confirmed diagnosis of CF who were age 17 years or older, chronically infected with *Pseudomonas aeruginosa*, and treated for PEx with intravenous antibiotics at least once between June 1, 2009 and June 30, 2012. Patient follow-up ended on June 30, 2012 and they were censored if they died or underwent lung transplantation prior to June 30, 2012.

The decision to initiate and terminate treatment with IV antibiotics was made by one of two treating physicians. Treatment for PEx with IV antibiotics was prompted by significant worsening respiratory or systemic symptoms, typically with an associated fall in FEV_1_. In concordance with the Cystic Fibrosis Foundation (CFF) guidelines [20], treatment for a PEx consisted of two intravenous antibiotics (e.g. beta-lactam or cephalosporin paired with an aminoglycoside or colistin) targeting *P. aeruginosa*, augmentation of chest physiotherapy, and continuation of all background therapies including inhaled antibiotics. We did not use susceptibility testing in selecting antibiotics due to its poor prediction of clinical response [23]. The antibiotics used most frequently were ceftazidime, piperacillin-tazobactam, meropenem, tobramycin and colistin.

**Fig. 1:**
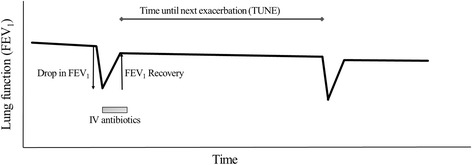
Hypothetical Course of Cystic Fibrosis Pulmonary Exacerbations. This figure illustrates the hypothetical course of changes in lung function (defined as FEV1 in this study) over time. Our main outcome of interest was time until next exacerbation (TUNE). For each exacerbation, we examined the “drop” in FEV1 (change in FEV1 from baseline at onset of exacerbation), “recovery” of FEV1 (change in FEV1 over the course of treatment of exacerbation) and duration of intravenous (IV) antibiotics as covariates

Patients were enrolled in the study at time of first PEx during the study period. Baseline age, gender, BMI, FEV_1_ and presence of CF-related diabetes were recorded at time of study entry. Longitudinal FEV_1_ data obtained from clinic visits and hospitalizations were used to determine pre-exacerbation baseline FEV_1_ and changes in FEV_1_ prior to and after treatment for a PEx. FEV_1_ was reported as percent predicted using reference values from Hankinson et al. [24]. Baseline FEV_1_ was defined as best FEV_1_ in the six months prior to the index PEx. For subsequent PEx the recovery FEV1 from the previous PEx was used as the new baseline. The recovery FEV1 was defined as the FEV1 measured after completion of IV antibiotics used to treat a pulmonary exacerbation. For each PEx, we examined drop in FEV_1_ at onset of PEx and recovery of FEV_1_ with treatment of the PEx (Fig. 1), (reported as an absolute percent change in FEV_1_). We also assessed whether patients had a “response” in FEV_1_ with treatment as a categorical variable (defined as recovery of 90% of FEV_1_ prior to PEx). Duration of IV antibiotics was obtained from registry data and reported in days.

### Statistical analysis

Statistical analysis was performed using SAS software, version 9.4 (SAS Institute, Cary, NC) and Excel (Microsoft, Redmond, WA). Baseline patient-level characteristics and time-varying exacerbation-level characteristics were reported using means and standard deviations for normally distributed continuous variable and median and interquartile range for non-normally distributed continuous variables. Categorical variables were reported using frequency and percent. Available-case analysis was employed when missing covariate data was present.

Multiple exacerbation episodes and subsequent time until next exacerbation (TUNE) were analyzed using Andersen-Gill counting process models, an extension of the Cox proportional hazard (PH) model for multiple failure outcomes [25, 26]. This approach models the hazard for an exacerbations event over the study period conditional on a set of covariates. The risk interval included the total duration of the study period and a clock-reset method was not used. A key assumption of the Andersen-Gill model is that the times between recurrent events are uncorrelated when conditioned on the time-varying covariates specified in a given model. Robust standard errors were used. Algebraically, the Andersen-Gill model is represented as: λik(t) = λ0(t)exp.{Xiβ} such that for a given patient i, λik(t) represents the hazard function for the kth event at time t, λ0 represents the common baseline hazards for all events of the study period, Xi represents the vector of covariates for an individual, and β represents a fixed vector of coefficients.

Recurrent exacerbations were also evaluated using Prentice, Williams and Peterson gap time (PWP-GT) models [27] which account for the ordering of events. We considered this model in addition to the Andersen-Gill model to explore the assumption that different exacerbation events can have differing baseline hazards and that covariate effects may differ across events. In this model, the time until next exacerbation includes the number of days from the last day of treatment of the previous pulmonary exacerbation to the first day of the subsequent pulmonary exacerbation. Algebraically, the PWP-GT model is similar to the Andersen-Gill model and is represented as λik(t) = λ0k(t)exp.{Xiβ}. Whereas λ0 represents common baseline hazards in the Andersen-Gill model, λ0k represents the event-specific baseline hazards for the kth event.

First, we performed a univariable analysis of potential demographic and PEx event-specific covariates. Fixed covariates included baseline age, baseline BMI, baseline FEV1 defined as the best FEV1 in the prior 6 months to enrollment, gender, and presence of CFRD. Time-varying covariates included duration of IV antibiotic treatment within an exacerbation, fall in FEV1 at onset of PEx from the recovery FEV1 from the previous PEx, recovery of FEV1 with treatment of PEx and whether 90% of pre-exacerbation FEV1 was recovered with treatment of PEx. Continuous covariates were modeled using linear terms. We planned to build a final multivariable model to determine a final model of factors significantly associated with TUNE using model entry criterion of *P* ≤ 0.1 with manual stepwise selections. We also evaluated a fully adjusted model in which all covariates of interest were evaluated in a single multivariable model as well as separate models for each time-varying covariate with adjustment for all baseline covariates. We considered separate adjusted models for each time-varying covariate in addition to the fully adjusted model in order to further evaluate how adjustment for fixed baseline characteristics affected univariable estimates for each time-varying covariate.

Based on the results shown below that there was a lower risk of a pulmonary exacerbation with older age we performed a subgroup analysis in which differences in demographic and clinical characteristics between patients above and below the median age for the cohort were evaluated. Categorical variables were compared using Fisher’s exact test and continuous variables were compared using two-sample t-tests.

## Results

### Demographic data

**Fig. 2:**
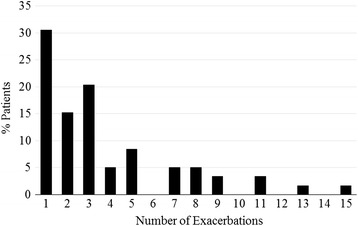
Distribution of Cystic Fibrosis Patients Pulmonary Exacerbation Frequency. Graph demonstrating percent of patients with given number of pulmonary exacerbations during the 3 year study period

We examined 59 unique patients who experienced a total of 221 CF pulmonary exacerbations requiring IV antibiotics which calculates to 3.8(sd = 3.3) exacerbations per patient over 3 year study period. No patients died during the study period and 4 patients underwent lung transplantation. At time of study entry, mean age was 28.2(sd = 6.8) years, mean baseline FEV_1_ was 62% (sd = 21%) predicted and mean BMI was 21.8(sd = 4.1) kg/m^2^ (Table 1). Subjects were 69% female and 49% Phe508del homozygotes. Eighteen subjects (30.5%) had only one PEx, the index exacerbation, during the three-year study period, and 66% of patients had three or fewer exacerbations (Fig. 2). Ninety-two percent (*n* = 203 of 221) of exacerbations included in the analysis had an FEV1 measured prior to the initiation of antibiotics and within 7 days of completing antibiotics.

**Table 1:** Selected Demographics and Comparison by Age Cohorts

	Entire Cohort	Age < 28 yrs	Age ≥ 28 yrs	*P*-value
Age at baseline, yrs	28.2 ± 6.8	22.9 ± 3.4	33.6 ± 4.9	–
BMI at baseline, kg/m^2^	21.8 ± 4.8	20.1 ± 3.2	23.4 ± 4.4	<0.01^†^
FEV_1_ at baseline, % predicted	62 ± 21	56 ± 21	60 ± 24	0.31^†^
Female gender	69%	77%	62%	0.27^‡^
Phe508del homozygote	49%^a^	57%	41%^a^	0.30^‡^
CF-related diabetes	10%	13%	7%	0.67^‡^
Pancreatic insufficiency	92%	100%	83%	0.02^‡^

Data are presented as % or mean ± standard deviation

*BMI* body mass index, *FEV1* forced expiratory volume in 1 s, CF cystic fibrosis. The three right-hand columns are the results of the subgroup analysis comparing subjects below and above the median age for the cohort

^a^Genotype missing for one patient

^†^ *P* value is computed using two-sample t-tests

^‡^ *P* value is computed using Fisher exact test

### Exacerbation characteristics

**Fig. 3:**
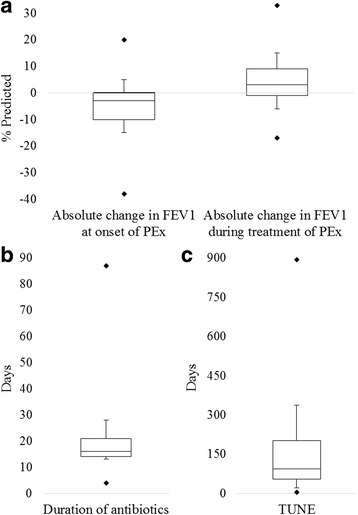
Characteristics of Pulmonary Exacerbations. Box and whisper plots with boxes showing median and interquartile range, lines showing 10th and 90th percentiles, and dots showing minimum and maximum of different characteristics of pulmonary exacerbations (PEx) in our cohort. **a** Median absolute change in FEV1 at onset of PEx (“drop” in FEV1 with PEx) was −3% predicted (interquartile range − 10 to 0% predicted, minimum −38% predicted, maximum 20% predicted). Median absolute change in FEV1 during treatment of PEx (“response” in FEV1 to IV antibiotics) was +3% predicted (interquartile range − 1 to +9% predicted, minimum −17% predicted, maximum 33% predicted). **b** Median duration of antibiotics was 16 days (interquartile range 14–21 days, minimum 4 days, and maximum 87 days). **c** Median time until next exacerbation (TUNE) was 93.5 days (interquartile range 54–203 days, minimum 4 days, and maximum 893 days)

The median duration of IV antibiotics was 16 days (Fig. 3), and 22% of PEx were treated with IV antibiotics for >21 days (*n* = 49 of 221). An additional course of IV antibiotics was required in the same patient within 30 days in 14% of PEx (*n* = 23 of 162).

The median absolute change from baseline in FEV_1_ at onset of PEx (drop in FEV_1_ with PEx) was −3% (relative change of 6%) predicted (Fig. 3; complete data in 202 PEx). The median absolute change in FEV_1_ with treatment of IV antibiotics (recovery in FEV_1_) was +3% predicted (relative change of 6%) (Fig. 3; complete data in 203 PEx). FEV_1_ recovered to within 90% of baseline in 58% of PEx (complete data in 201 PEx). Median time to next exacerbation (TUNE) was 93.5 days (Fig. 3; complete data in 162 PEx).

### TUNE hazard models

We then examined demographic, physiologic and PEx-related variables as predictors of TUNE using the Andersen-Gill model, which is a time to event analysis that accounts for recurrent events (i.e. previous PEx). Exploratory univariable analyses showed that for an increase in baseline age by 1 year, the HR for a PEx was 0.97 (*P* = 0.03, 95% CI 0.95–1.00) (Table 2). These results suggest that increasing age was associated with a lower risk for pulmonary exacerbations which is, contrary to some previous reports. The association between age and TUNE did not each statistical significance in the PWP-GT univariable model (*P* = 0.19, HR 0.96, 95% CI 0.96–1.01) (Table 2). None of the remaining examined covariates met criteria for stepwise entry into a multivariable model in either the Andersen-Gil of PWP-GT models. Specifically, fall in FEV_1_ at onset of PEx, recovery of FEV_1_ with treatment and duration of IV antibiotics were not associated with TUNE in our cohort.

**Table 2:** Time until Next Exacerbation (TUNE) Univariable Models

Variable	Andersen-Gill		PWP-GT*	
	HR (95% CI)	*p*-value	HR (95% CI)	*p*-value
Age at baseline	0.97 (0.95–1.00)	**0.03**	0.98 (0.96–1.01)	0.19
BMI at baseline	0.99 (0.95–1.03)	0.52	0.99 (0.96–1.02)	0.61
FEV_1_ at baseline	1 (0.96–1.03)	0.81	1 (0.99–1.00)	0.33
Male gender	0.75 (0.52–1.09)	0.13	0.86 (0.68–1.09)	0.20
CF-related diabetes	0.81 (0.48–1.37)	0.42	1.01 (0.7–1.46)	0.94
Duration of IV antibiotics	0.93 (0.98–1.02)	0.93	1.01 (0.99–1.02)	0.46
Absolute change (“drop”) in FEV_1_ with onset of exacerbation	1 (0.99–1.01)	0.64	1 (0.98–1.01)	0.65
Absolute change (“recovery”) in FEV_1_ with treatment	1.01 (0.99–1.03)	0.43	1 (0.98–1.02)	0.80
Recovery to 90% of baseline FEV_1_	0.77 (0.53–1.11)	0.16	0.95 (0.73–1.23)	0.67

No final models were created for either method due to no more than a single co-variate reaching the *p*-value required for model entry

*BMI* body mass index, *FEV*_*1*_ forced expiratory volume in 1 s, CF cystic fibrosis, *HR* hazard ratio (95% confidence intervals reported in parentheses), *PWP-GT* Prentice, Williams and Peterson gap time

In a fully adjusted Andersen-Gill model that included all covariates of interest, baseline age, baseline FEV1, and male gender were significantly associated with having an exacerbation event. In a separate adjusted Andersen-Gill model in which the duration of IV antibiotics was modeled with adjustment for all fixed baseline covariates, baseline age remained significantly associated with having an exacerbation event (*p* = 0.04) and marginally significant in all other models (*p* < 0.10). As with previous models fall in FEV1 at onset of PEx, recovery of FEV1 with treatment and duration of IV antibiotics were not associated with TUNE.

### Analysis by age cohorts

Since age was associated with TUNE in multiple models which suggested, we decided to more closely examine the paradoxical association of older age and decreased risk of PEx. We stratified our cohort by median age (28 years) and compared the two groups across several variables (Table 1). Mean age for the younger cohort was 22.9(sd = 3.4) years, and mean age for the older cohort was 33.6(sd = 4.9) years. The older cohort had a higher mean baseline BMI (23.4 vs 20.1 kg/m^2^, *P* < 0.01) and were less likely to be pancreatic insufficient (83% vs 100%, *P* = 0.02) compared to the younger cohort. The baseline mean FEV_1_ was not statistically different despite a mean age difference of more than 10 years between the cohorts. Additionally the prevalence of CF-related diabetes and F508del homozygous genotype were lower in the older cohort (Table 1).

## Discussion

In this analysis of PEx in adult CF patients infected with *P. aeruginosa*, we show that the change in FEV_1_ at onset of PEx, the degree of FEV_1_ recovery in response to treatment and duration of IV antibiotics are not associated with TUNE. Further, we detected a trend towards a paradoxical relationship between age and TUNE with older age being associated with reduced hazard of TUNE.

The primary goal in treating PEx is the resolution of symptoms and improvement of lung function back to baseline. An additional less proximate goal is to extend the time before PEx treatment will be needed again. This is an important endpoint because of the significant morbidity associated with each PEx, risk of failure to recover lung function following a PEx and short-term as well as cumulative toxicity of antibiotics used to treat PEx [1, 3–8]. Several studies have assessed PEx risk factors and consistently the strongest predictor for future PEx is the patient’s previous history of PEx frequency [16, 17, 22]. We controlled for this in our statistical analysis which allowed us to assess the impact of additional variables. Other factors that have been associated with increased PEx frequency relate to the severity of lung disease which include lower baseline FEV_1_, greater use of chronic pulmonary therapies and co-morbidities such as CFRD and liver disease [15, 16].

The data on the impact of acute spirometric changes associated with PEx and treatment duration on TUNE are less robust and conflicting. Two previous studies found no relationship between duration of IV antibiotics and TUNE [15, 16]. Another study actually found a shorter TUNE with a longer course of IV antibiotics [22]. Vandevanter et al. have reported that either a very short course or a longer course are associated with shorter TUNE [16, 17]. There is also conflicting data on the relationship between TUNE and FEV_1_ fall at antibiotic initiation as well as with degree of FEV_1_ recovery post PEx treatment. Similar to our data, one study failed to detect a relationship between FEV_1_ drop at time of PEx identification and TUNE [22]. Interestingly and somewhat paradoxically, the same study reported a longer TUNE with a failure to recover to FEV_1_ baseline [22]. In contrast Sequeiros et al. found no relationship between FEV1 recovery and TUNE, similar to our findings [15].

The reasons for the discrepant results from the various studies are not clear, but in part may be related to heterogeneity of the patient populations studied. Both the Vandevanter and Heltsche analyses included a significant number of patients under the age of 18 who were treated at multiple CF centers and also did not limit patients to those with chronic *P. aeruginosa* infection [16, 17, 22]. Our study and the analysis by Sequeiros analysis included only adult patients with *P. aeruginosa* infection treated at a single CF center and showed similar results [15]. It is likely that factors such as age, local treatment philosophy and airway microbiome impacts risk, treatment and response to PEx. This is consistent with CFF registry and other data that PEx treatment varies considerably across accredited centers [28, 29].

Our data provide support to the idea that TUNE is not significantly impacted by co-variates associated with a prior PEx. This has important implications for identification and management of PEx. Identifying and treating PEx early is an important goal as it has been shown that the greater the fall in FEV_1_ from baseline, the lower the likelihood of returning back to baseline FEV_1_ [6]. Previous data suggest that FEV_1_ plateaus at day 9 in 90% PEx patients [21] and there is no change in symptom improvement between PEx patients treated for 10–14 days or more than 14 days [22]. This suggests that extending the course of treatment beyond 14 days is not likely to have significant symptom or FEV_1_ benefit in the vast majority of patients. Further our data and others’ suggest that extending the duration of antibiotics in an effort of preventing a future PEx is also not likely to succeed [30]. This is an important observation since up to 30% of PEx are treated with more than 14 days of antibiotics [30, 31]. There is the potential however of incurring acute and cumulative toxicity [19] as well as inducing bacterial antibiotic resistance [32] which has been associated with increased morbidity and mortality [18, 33].

In contrast to previous findings and somewhat paradoxically [15], older age was associated with a longer TUNE in our adult cohort in results from the Andersen-Gill model when exacerbations are treated independently. We suspect that this may be due to a “healthy survivor” effect which is suggested by the observation that FEV1 decline is slower in patients over the age of 25 compared to patients between the ages of 12–24 [34, 35]. Our analysis of the cohorts above and below the median age of our study population supports this hypothesis. Despite being more than 10 years older on average, the older cohort had markers of milder disease, including higher baseline BMI and higher prevalence of pancreatic sufficiency. The older half of the cohort also had a trend toward slightly higher baseline FEV1 and slightly lower rate of F508del homozygote genotype. Further supporting the idea of a healthy survivor effect is the observation from a prior study that the frequency of PEx increases until the age of twenty-five and decreases thereafter [17]. Nonetheless, we recognize that this paradoxical association may be due to other unmeasured variables though in our initial models we controlled for most of the variables known to impact pulmonary function and exacerbation risk in CF.

One limitation of this study is the retrospective analysis with its inherent concerns about the accuracy of the data. It should be pointed out, however, that our data includes exact dates for the beginning and ending dates of the antibiotics and matching lung function data. This allowed us to ascertain the dynamic FEV_1_ changes associated with PEx as well as duration of antibiotics with reasonable accuracy.

Another limitation of the study is the relatively small changes in fall and recovery of FEV1 associated with pulmonary exacerbations. We think this likely reflect the real-world setting of our analysis and the absence of fixed criteria for beginning or ending a course of IV antibiotics. Mitigating this concern is that all decisions with respect to when to begin antibiotics and their duration were made by one of the same two experienced physicians at a single center. In addition, the average number of PEx treated with IV antibiotics each year at our center has not changed over the past 10 years. This suggests that decisions to initiate and finish IV antibiotics have not significantly changed over the time frame of this study. Lastly, we recognize the limitations of available-case analysis such that different analyses may be based on different subsets of the data; however, we chose this method to fully utilize the available data in our sample and also note that patterns of missingness were similar across covariates.

## Conclusions

Changes in FEV_1_ associated with CF pulmonary exacerbation or treatment, including drop in FEV_1_ at onset of exacerbation and magnitude of FEV_1_ recovery were not predictive of time until next exacerbation. In addition, our cohort did not demonstrate any association of duration of antibiotic therapy with TUNE. However, older age may be associated with a longer TUNE, likely due to a healthy survivor effect.

## Abbreviations

BMI: Body mass index
CF: Cystic Fibrosis
CFFPR: Cystic Fibrosis Foundation Patient Registry
CFRD: Cystic Fibrosis-related diabetes mellitus
FEV1: Forced expiratory volume in 1 s
HR: Hazard ratio
IV: Intravenous
PEx: Pulmonary exacerbation
TUNE: Time until next exacerbation
